# Thymoline Soap

**Published:** 1879-12

**Authors:** 


Thymoline Soap is a soap designed especially for the antiseptic cleansing of physicians' instruments. The soap contains a sufficient quantity of the disinfectant, and the odor of the thymoline is by no means unpleasant. In this day, when so much is said respecting the transmission of disease by surgical instruments, it will be a satisfaction to make use of an agent in cleansing them, which will, at least, assist in their disinfection.—*Chicago Medical Journal and Examiner.*

